# Strontium Ions Substitution in Brushite Crystals: The Role of Strontium Chloride

**DOI:** 10.3390/jfb2020031

**Published:** 2011-05-31

**Authors:** Mohammad H. Alkhraisat, Carmen Rueda, Enrique López Cabarcos

**Affiliations:** Departamento de Química Física II, Facultad de Farmacia, UCM, 28040 Madrid, Spain; E-Mails: crueda@farm.ucm.es (C.R.); cabarcos@farm.ucm.es (E.L.C.)

**Keywords:** Brushite, calcium phosphate cement, strontium, glycolic acid

## Abstract

The incorporation of strontium chloride to brushite cement was successful to introduce strontium ions within the lattice of brushite crystals. The effect of strontium ions on brushite cement properties was concentration dependent; such that, the addition of 5% and 10% (w/w) SrCl_2_ significantly increased the cement FST and the addition of 10% SrCl_2_ decreased the cement tensile strength. Further, cement weight loss was shown to be increased by cement modification with SrCl_2_. The combination of ionic substitution and the degradability of brushite cements would constitute a system for the local delivery of strontium ions in the treatment of osteoporosis.

## 1. Introduction

Osteoporosis results from reduced bone mass and disruption of the micro-architecture of bone, giving decreased bone strength and increased risk of fracture, particularly of the spine, hip, wrist, humerus, and pelvis. Epidemiologically, fractures caused by osteoporosis affect one in two women and one in five men over the age of 50, resulting in an estimated annual cost to the health services of around 30 billion Euros in Europe with increased risk of disability and mortality [1,2].

Treatment of osteoporosis with strontium containing compounds was reported about 40 years ago [3,4]. Nowadays, a commercial strontium containing drug has been used clinically and shown to reduce vertebral and non-vertebral (including hip) fractures in postmenopausal women with osteoporosis [5,6]. The beneficial effects of strontium stem from the prevention of bone loss. *In vitro* studies showed that strontium ions (Sr^2+^) decreased the differentiation and resorping activity of osteoclasts as well as increased osteoclast apoptosis [7,8,9]. Meanwhile, Sr^2+^ was shown to enhance preosteoblastic cell proliferation and collagen synthesis [10]. Consequently, Sr^2+^ ions depress bone resorption and maintain bone formation.

Strontium-based and zinc-based ionomeric cements were tested for their osteoconductive properties [11] and the results indicated that ionomeric cement with higher Sr content was the most osteoconductive. Leroux *et al.* synthesized calcium strontium hydroxyapatites based on ionic calcium phosphate cements [12]. The presence of a significant amount of NO^3−^ containing impurities prevents its further application. Therefore, novel Sr-containing hydroxyapatite cement with no impurities in its final product was developed [13].

Substitution of calcium by strontium within amorphous calcium phosphate, apatitic calcium phosphate, hydroxyapatite, octacalcium phosphate and dicalcium phosphate dihydrate (DCPD) was studied [14]. It was reported that calcium substitution by strontium in DCPD was the most efficient, due to the fact that all Ca sites in the DCPD lattice can be occupied by Sr atoms [14].

Subsequently, brushite (DCPD) cement has the advantage of being resorbable [15], strontium substitution for calcium is more efficient [14] and the set brushite cement does not have harmful impurities [15]. For this reason, brushite cement set with 1M glycolic acid was modified by the addition of 1%, 2%, 5%, 8%, and 10% (w/w) SrCl_2_. Thereafter, the effect of the added SrCl_2_ on cement microstructure, final setting time, cohesion, diameter tensile strength and morphology was studied.

## 2. Materials and Methods

### 2.1. Brushite Cement Synthesis

DCPD, monocalcium phosphate (MCP), calcium carbonate (CC), sodium pyrophosphate, glycolic acid (from Sigma-Aldrich) and strontium chloride (SrCl_2_) (from Merck) were used without further purification. Beta-tricalcium phosphate (β-TCP) was synthesized by heating a stoichiometric mixture of CC and DCPD at 900 °C for 14 hours (Eq. (1)). Formation of pure β-TCP was assured by X-ray diffraction analysis.

(1) $$\text{CaC}O_{3}+2\text{CaHP}O_{4}\cdot 2H_{2}O\to Ca_{3}{\left(PO_{4}\right)}_{2}+5H_{2}O+CO_{2}$$

The cement solid phase made of β-TCP (1.428 g), MCP (0.8 g) and sodium pyrophosphate (0.012 g) was modified with 1%, 2%, 5%, 8%, 10% (w/w) SrCl_2_. The β-TCP/ MCP molar ratio of 1.35 was selected to obtain an excess of β-TCP that improves cement compatibility and slows down the resorption rate of the cement *in vivo* [15,16].

Cement liquid phase was 1M glycolic acid aqueous solution. The cement setting reaction was induced by mixing the solid phase with the liquid phase using a spatula over a glass slab for 30 s, in a powder to liquid ratio (P/L) of 2.5. The cement setting reaction occurs as follows (Equation 2):

(2) $$\beta -Ca_{3}{\left(PO_{4}\right)}_{2}+\text{Ca}{\left(H_{2}PO_{4}\right)}_{2}+8H_{2}O\to 4\text{CaHP}O_{4}.2H_{2}O$$

### 2.2. Preparation of Cylindrical Brushite Cement Samples

The cement paste resulting from mixing the liquid and solid phases was used to fill one-face-opened polyethylene cylinders with an internal diameter of 10 mm and a height of 5 mm. The cement sample volume (∼392.5 mm^3^) and the exposed cement surface (∼78.5 mm^2^) were kept constant for all specimens.

### 2.3. Measurement of Cement Final Setting Time and Diametral Tensile Strength

The cement setting time was measured according to the international standard ISO1566 for dental zinc phosphate cement. According to this method, the cement is considered set when a 400 g weight loaded onto a Vicat needle with a tip diameter of 1 mm fails to make a perceptible circular indentation on the cement surface. New cement samples (10 mm in Φ, 5mm thick) were aged in double-distilled water at 37 °C for 24 h prior to testing. Wet diametral tensile strength (DTS) was measured on Pharma Test PTB 311 and calculated from the failure load applied along the diametral plane of the samples (N = 5).

### 2.4. Particle Release from Cement Surface

The cement cylinders were left to set at room temperature and humidity. Afterwards, each sample was immersed in 5 mL of distilled water at 37 ± 1 °C for 24 hours in a thermostatic bath under constant stirring (70 rpm). Three specimens were prepared for each combination of liquid/solid phase. After 24 h of incubation, the cement particles released to the liquid media were collected using a Millipore membrane (Millipore Ibérica S.A, Madrid, Spain) with a pore size of 0.10 μm and analyzed by optical microscopy (Motic, Barcelona, Spain). Then, the Millipore membranes and cement cylinders were dried and weighed. The percentage of weight loss was calculated from the cement specimen dried weight (Cw) and the released particles dry weight (Pw) as follows:

(3) $$\text{Solid weight loss}\left(\%\right)=\frac{Pw}{Pw+Cw}100\%$$

### 2.5. X-ray Diffraction of Brushite Cements

Rietveld analysis of the set cements diffraction patterns obtained by X-ray diffraction using a Philips X'pert diffractometer (Cu-*K*_a_ radiation, 45 kV, 40 mA) was done. Data were collected in the interval between 2θ = 10° and 80° with a step size of 0.03°, and a normalized count time of 3 s/step. The mineral composition of the cement was checked by means of structural model files of brushite (ICSD 016132) and β-TCP (ICSD 06191).

## 3. Results

The effect of SrCl_2_ on the cement setting reaction was concentration dependent as indicated by the measurement of the cement FST. Such that the addition of low concentration of SrCl_2_ did not significantly affect the cement FST, whereas concentrations of 5% and 10% (w/w) SrCl_2_ increased the cement FST to *ca.* 20 and 45 minutes, respectively (Table 1). Moreover, it was observed that the cement paste was more liquid-like with the increase in SrCl_2_ concentration.

**Table 1:** Strontium effect on brushite cement final setting time.

**% SrCl_2_ (w/w)**	**FST (minutes)**
0	6.9 ± 0.3
1%	7.1 ± 0.1
5%	19.1 ± 0.2
10%	45 ± 1

**Figure 1:**
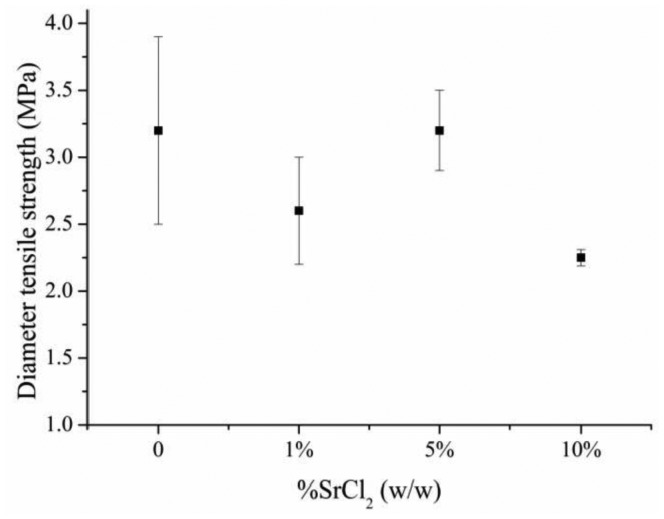
The effect of strontium ions in brushite cement tensile strength.

The presence of Sr^2+^ in the cement paste seems to affect the cement diameter tensile strength. Unmodified brushite cement had a diameter tensile strength of 3.2 ± 0.7 MPa. SrCl_2_ concentrations up to 5% did not significantly affect the cement tensile strength. Nevertheless, the addition of 10% (w/w) SrCl_2_ decreased the cement tensile strength to *ca.* 2 MPa (Figure 1).

**Figure 2:**
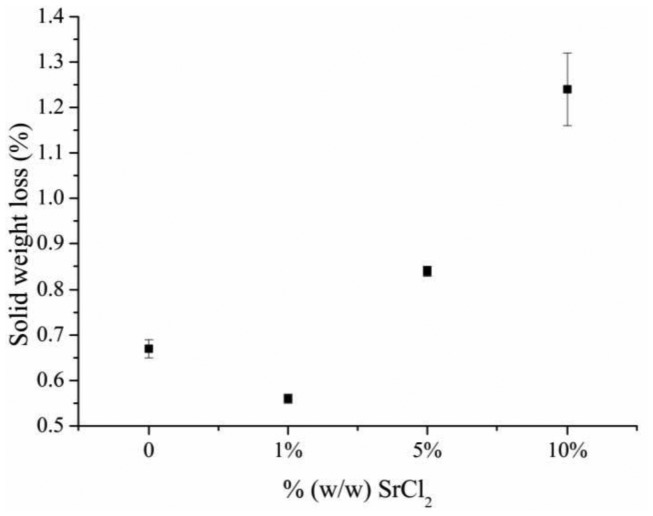
Strontium ions effect on brushite cement cohesion.

**Figure 3:**
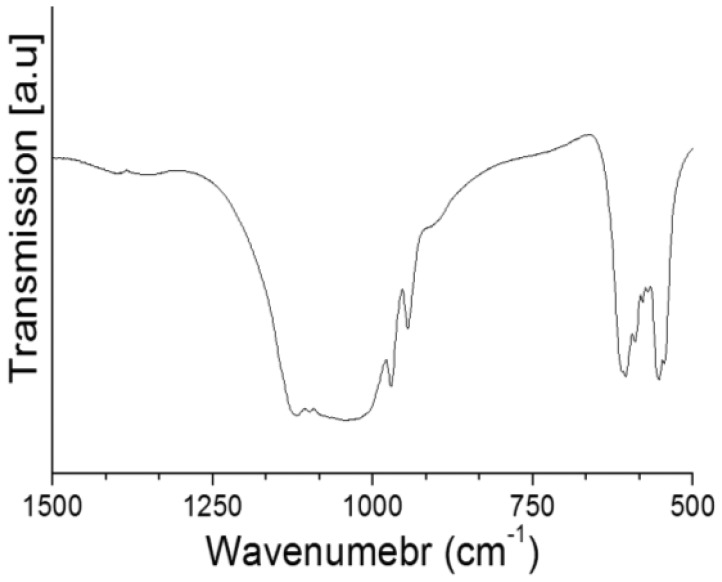
FTIR spectrum of the particles released from cement surface.

Herein, the cement solid weight loss was used as an indicator for cement cohesion as reported in previous studies [17,18,19]. The cement weight loss was generally increased with the increase in SrCl_2_ concentration (Figure 2). The solid weight loss of Sr-free cements was about 0.5% that was increased to *ca.* 0.9 and 1.7% for cements prepared with SrCl_2_ of 5 and 10%. The released particles were β-TCP as indicated by FTIR spectroscopy (Figure 3).

**Figure 4:**
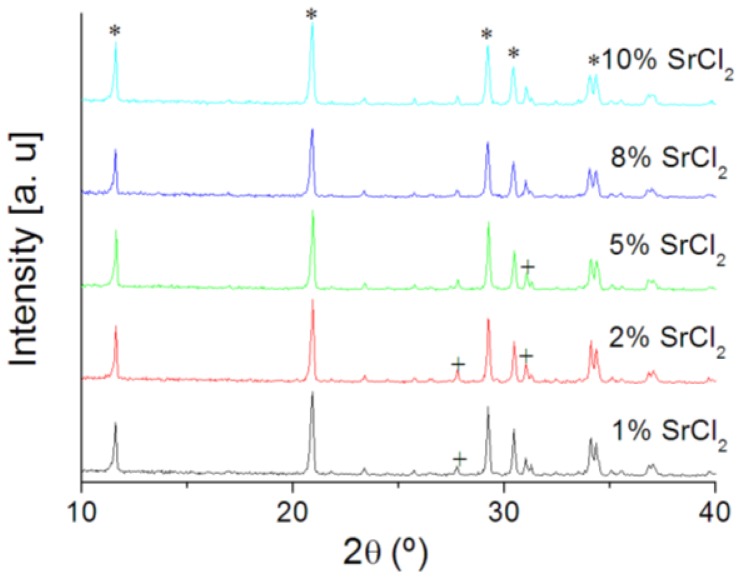
X-ray diffraction patterns of brushite cements modified by SrCl_2_. Predominant peaks of brushite (*) and β-TCP (+) are labeled.

**Figure 5:**
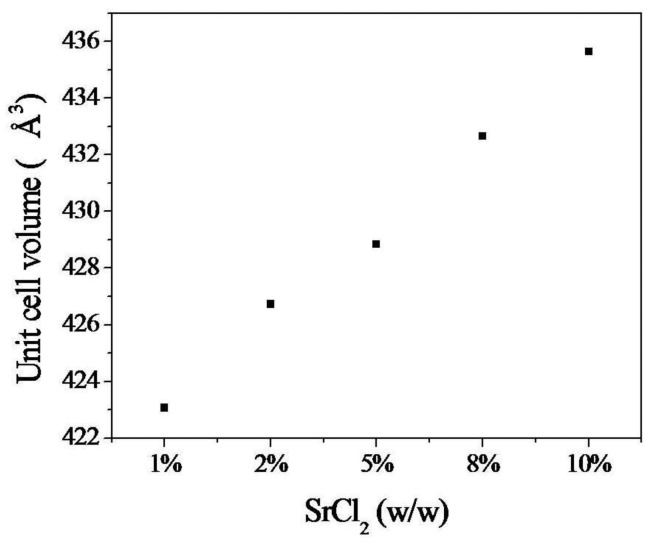
Cell unit volume of Sr-modified brushite cements.

Rietveld analysis of the XRD spectra of cement samples was summarized in Table 2. The cement matrix were composed of β-TCP in a predominantly brushite matrix (Figure 4). The diffraction peaks of brushite are shifted toward lower diffraction angles indicating an increase in unit cell volume. The relative amount of brushite in cement matrix did not seem to be affected by the increase in SrCl_2_. Rietveld refinement of brushite unit cell parameters indicated a conspicuous increase in the parameters value with the increase in strontium concentration up to 8% (w/w). However, the unit cell parameters *a*, *c* decreased while the *b* increased at 10% (w/w) SrCl_2_ (Table 2). Nevertheless, the unit cell volume calculated for monoclinic system increased linearly for all SrCl_2_ concentrations (Figure 5).

**Table 2:** Lattice parameters of brushite crystals in set cement.

**% SrCl_2_ (w/w)**	**Cement composition**		**Lattice parameters (Å)**		
	**DCPD (%)**	**β-TCP (%)**	***a***	***b***	***c***
1	83	17	6.365250 ± 0.002	15.187980 ± 0.006	5.812983 ± 0.003
2	81	19	6.367848 ± 0.002	15.191320 ± 0.006	5.814205 ± 0.002
5	82	18	6.369741 ± 0.001	15.192880 ± 0.004	5.815243 ± 0.002
8	83	17	6.376474 ± 0.004	15.196140 ± 0.008	5.818215 ± 0.003
10	87	13	6.375908 ± 0.002	15.204630 ± 0.006	5.818039 ± 0.003

## 4. Discussion

The unit cell of DCPD contains four calcium and four phosphorus atoms. The Ca and P atoms are linked to form corrugated sheets where each two sheets are joined together by water molecules. All Ca sites in the crystal lattice are characterized by almost identical coordination [14]. As all Ca sites in the DCPD lattice can be occupied by Sr atoms, brushite cements could be used as a delivery system for strontium ions.

Strontium ions were successfully incorporated in the structure of brushite crystals as evidenced by an increase of unit cell volume with the increased strontium content (Figure 5). This is related to the fact that the ionic radius of Sr^2+^ (1.13 Å) is larger than calcium ion (1.00 Å).

Brushite cement FST was increased significantly at high SrCl_2_ concentration (Table 1). The dissolution of the basic β-TCP is determinant to brushite cement FST. Christoffersen *et al.* reported that Sr^2+^ inhibits the rate of dissolution of hydroxyapatite, similarly, the presence of Sr^2+^ in the cement paste could inhibit the dissolution rate of β-TCP increasing the cement FST [20]. Strontium ions were also reported to inhibit apatite deposition and retard calcium phosphate precipitation. Thus, Sr^2+^ could inhibit brushite crystal growth [21] and increase the cement FST.

The solid particles released from the brushite cements were β-TCP. Cement solid weight loss was used to indicate brushite cement cohesion [17,18,19]. Brushite solubility affects the cement cohesion as reported in a previous study [17]; such that the more soluble the brushite crystals are, the more β-TCP particles are released. It was reported that strontium substitution for calcium expands hydroxyapatite crystals lattice and increases their solubility [20]. Herein, strontium incorporation causes brushite crystals to expand and thus decreases their stability, resulting in more soluble brushite crystals. Consequently, β-TCP particles release and cement solid weight loss increased.

Brushite cement diameter tensile strength was significantly affected when 10% (w/w) SrCl_2_ was added. At this concentration, strontium content within brushite lattice was the highest as indicated by cell unit volume. This high content of strontium may decrease the microhardness of brushite crystals and, therefore, the cement diameter tensile strength. Cement porosity is determinant to the cement mechanical properties. The increased solubility of Sr-containing brushite crystals could increase the number and/or the size of cement pores and thus decrease cement tensile strength.

## 5. Conclusions

The modification of brushite cements with strontium chloride is efficient to induce calcium ions substitution by strontium ions.
